# DON’T WORRY, BE HAPPY NOW, PET OWNERS: THE RELATION BETWEEN PET OWNERSHIP AND ANXIETY AND DEPRESSION IN LATE LIFE

**DOI:** 10.1093/geroni/igz038.589

**Published:** 2019-11-08

**Authors:** Courtney J Bolstad, Carolyn E Adams-Price, Michael R Nadorff

**Affiliations:** Mississippi State University, Mississippi State, Mississippi, United States

## Abstract

Pets can provide older adults a means of social support, which can combat problems faced in later life including loneliness, anxiety, and depression. However, current research findings in this area are mixed. The current study explored the differences in anxiety and depression between pet owners and non-pet owners and how pet ownership was associated with these symptoms after accounting for other established correlates. We hypothesized pet owners would endorse fewer symptoms of anxiety and depression than non-pet owners and owning a pet would be associated with these symptoms even after accounting for other common correlates. Participants included 608 older adults aged 70 to 95 that were included in the University of Alabama at Birmingham Study of Aging. As hypothesized, results indicated that pet owners endorsed significantly fewer symptoms of anxiety and depression than non-pet owners. Hierarchical regressions indicated that owning a pet explained a significant amount of variance in anxiety symptoms even after controlling for depression, self-reported health, and demographics. However, owning a pet did not have a significant association with depressive symptoms after accounting for anxiety, self-reported health, and demographics. These results suggest that lower rates of anxiety and depression are related to owning a pet and that pet ownership is associated with fewer anxiety symptoms, but not depressive symptoms, independent of several established correlates of anxiety. Future research is needed to determine the specific mechanisms of pet ownership that comprise this relationship as well as whether pet ownership may longitudinally reduce or buffer against anxiety in late life.

